# Urban–rural disparity in risky sexual behavior, HIV knowledge, and healthy practices among men who have sex with men: A cross-sectional study in Southeast China

**DOI:** 10.1371/journal.pone.0312006

**Published:** 2024-11-11

**Authors:** Wanjun Chen, Lin Chen, Lin He, Chengliang Chai

**Affiliations:** Department of AIDS and STD Prevention and Control, Zhejiang Provincial Center for Disease Control and Prevention, Hangzhou, Zhejiang, China; Centers for Disease Control and Prevention, UNITED STATES OF AMERICA

## Abstract

To identify differences in human immunodeficiency virus (HIV) risky behavior and healthy practices between rural and urban men who have sex with men (MSM) in Southeast China, **a** cross-sectional study was conducted on MSM aged ≥ 18 years recruited from four community-based organizations (CBOs) in seven cities in Zhejiang Province between October 2022 and March 2023. An electronic survey questionnaire was completed by the study participants to collect HIV risky behavior and healthy practices. The chi-square test and multiple logistic regression were used for analysis. Among the 1,993 MSM participants, 21.1% (420) were rural MSM. Compared with urban MSM, rural MSM was more likely to have a low level of education (high school and below; 46.2% vs. 40.2%, p = 0.028) and a low annual income (≤ 90,000 RMB; 71.2% vs. 64.7%, p = 0.013). Multiple logistic regression showed that, compared with urban MSM in the past 6 months behaviors, rural MSM had lower odds of finding casual sex partners on the Internet (adjusted odds ratio [aOR]: 0.790, 95% confidence interval [CI]: 0.633–0.986), using stimulants with online casual sex partners (aOR: 0.688, 95% CI: 0.506–0.936), and high odds of ever having sex with a female (aOR: 1.460, 95% CI:1.036–2.058). Rural MSM reported lower odds of an HIV knowledge score of 2–3 (aOR: 0.586, 95% CI: 0.454–0.756), HIV testing more than twice (aOR: 0.583, 95% CI: 0.455–0.748), and obtaining four to five types of HIV information (aOR: 0.661, 95% CI: 0.529–0.826), as well as higher odds of high/very high awareness of HIV infection risk (aOR: 2.312, 95% CI: 1.638–3.263), compared to urban MSM. Rural MSM and urban MSM reported discrepancies in HIV risky behavior, HIV knowledge and HIV-related healthy practices. Paying more attention to HIV risky behavior and improving healthy practices in rural areas may help to prevent HIV transmission.

## Introduction

Gay men, bisexual men, and other men who have sex with men (MSM) are more likely to experience severe human immunodeficiency virus (HIV) infection, with prevalence rates of nearly 7% globally (not including the Sahara area) and 8% in China [1, 2]. Over the most recent 5-year period, 8% of MSM in Zhejiang Province had contracted HIV according to surveillance data, and 40% of newly diagnosed patients contracted HIV via male–male sexual contact [3]. The Joint United Nations Programme on HIV/AIDS established 95-95-95 targets, and intervention efforts have been conducted for MSM with some success [4]; the new infection rate has decreased in recent years [5]. However, HIV mitigation efforts in MSM require renewed focus. Specifically, new developments in technology have provided the means to monitor this virus better, and the recorded HIV prevalence rate in MSM from rural areas has shown a sharper increase than at any previous time [6].

A study in Zhejiang Province showed that HIV prevalence among MSM living in rural and urban areas was 3.9% and 24.2%, respectively [7]. Although the prevalence rate compared to urban MSM is lower in rural MSM, there has been a steady increase in HIV transmission in the latter group.

According to the knowledge–practice–attitude theory, HIV knowledge, HIV risky behavior, and attitudes toward HIV are related to HIV infection [8]. For MSM from rural areas, HIV risky behaviors have changed in recent years; for example, an increase in web-based casual sex behavior has been seen in this group [9–12]. This has been attributed to the availability of transportation to rural areas; the development of the Internet, especially social applications; and ever-expanding social and sexual networks [13].

HIV prevention program funding has tended to focus on urban areas because MSM are more likely to reside there [14]. For MSM, HIV prevention services are provided mainly by community-based organizations (CBOs), which tend to be located in urban centres [8, 13]. Fewer services are available in rural settings due to the low density of residents, as well as the lower prevalence of HIV infection, such that services are deemed less cost-effective [7, 15]. However, recent events and advances in technology are drawing people back to rural environments [16]. Therefore, the status of HIV prevention services in rural areas, and the differences between rural and urban MSM, need more attention.

Rural MSM would benefit from the accessibility to prevention services, key information, and knowledge provided by the Internet. Gaps in HIV infection-related behavior and prevention service accessibility between rural and urban MSM have narrowed; however, the differences between the two groups are unclear. It is important to understand the status of the two groups and their differences to more accurately evaluate the HIV infection risk for rural MSM and achieve proper implementation of mitigation measures.

Since 2022, we have conducted the HIV Behavior Cohort Survey (HBCS) in Southeast China, which has low HIV infection rates. The study targets MSM to identify the changes in characteristics over time of this group, in an attempt to identify the independent factors affecting their behavior from 2022 (inception) to 2025. The cohort study was conducted in seven cities in Zhejiang Province, with a sample size of approximately 2,000.

In this research, to compare the risky behavior of rural and urban MSM, and to determine health practices implemented to avoid HIV infection, we utilized the baseline data from the HBCS. The results may be useful for improving and scientifically implementing effective HIV prevention strategies.

## Materials and methods

### Study population and recruitment

Our cross-sectional study examining MSM at risk for HIV infection was conducted between October 1, 2022 and February 19, 2023, in Zhejiang Province. Surveys were conducted in Hangzhou, Ningbo, Jiaxing, Jinhua, and other cities, which together include more than 50% of all MSM in the province. MSM were eligible to participate if they met the following criteria: aged ≥ 18 years, HIV negative, had engaged in anal or oral sex with a man, and provided electronic informed consent.

### Survey procedures

Recruitment was conducted by four local CBOs using convenience sampling. Recruitment advertisements were placed in bars, parks, bathrooms, and dating application chat groups (Blued/WeChat/Tencent). Electronic questionnaires were designed to collect information on HIV knowledge, risky sexual behavior (condom use, stimulant use, web-based casual sexual behavior, etc.) and healthy practices (HIV testing, post-exposure prophylaxis [PEP], pre-exposure prophylaxis [PrEP], etc.). Participants were asked to scan a two-dimensional code and to complete the electronic questionnaire after signing the electronic informed consent form. Face-to-face- and telephone-based instructions regarding the questionnaire were provided by the CBOs. Participants were given a gift worth 30 RMB (USD 5), and 70 RMB (USD 12) was awarded to the CBOs for each participant recruited.

In total, 2,061 MSM met the recruitment criteria; of these, 12 were excluded (2 refused to take part in the study and 10 were HIV-positive). Thus, 2,059 MSM completed the questionnaire. However, 56 MSM were excluded (53 duplicates, determined by checking telephone numbers, and 3 with incomplete key information). A total of 1,993 participants were thus ultimately enrolled in the study. The response and effective response rates were 99.9% (2,049/2,061) and 97.3% (1,993/2,049), respectively.

### Definitions

One question was asked on residence: “Is your current permanent residence in an urban or rural area?”. Subjects generally considered townships and villages as rural areas, whereas cities and counties were considered to be urban areas.

Three questions were used to evaluate HIV knowledge, as follows. Question 1: “Which behavior has the highest risk of HIV infection?”. Answer options included ‘sex with a female virgin,’ ‘anal sex with a female,’ ‘anal sex with a male’ (correct), and ‘don’t know.’ Question 2: “Which sex role is more likely to lead to infection with AIDS?”. Answers options included ‘insertive,’ ‘acceptive’ (correct), ‘both,’ and ‘don’t know.’ Question 3: “Does the use of stimulants, such as rush poppers or zero capsules, increase the risk of HIV infection?”. Answer options included ‘yes’ (correct), ‘no,’ and ‘don’t know.’ A score of 1 point was given for answering a question correctly; thus, the total HIV knowledge score ranged from 0–3 points.

Two questions were asked to evaluate finding casual sex partners online or offline, as follows. Question 1: “Have you ever used the Internet to find casual sex partners on dating applications, in chat rooms, etc.?” Question 2: “Have you ever found casual sex partners in bars, parks, bathing pools, or other places?”

Stimulants in this study refer to rush poppers and zero capsules, which are commonly used by MSM.

There were five answer options for condom use: ‘never,’ ‘not frequently,’ ‘half the time,’ ‘frequently,’ and ‘every time.’ Condom use every time was classified as ‘consistent’; otherwise, it was categorized as ‘inconsistent.’

One question was asked to evaluate the types of HIV-related information obtained. Answer options included ‘PEP,’ ‘PrEP,’ ‘condom use,’ ‘HIV self-testing,’ and ‘serosorting.’ One point was given for each of the five options used, such that the total score ranged from 0–5 points.

### Statistical analysis

We used Microsoft Excel 2016 (Microsoft Corp., Redmond, WA, USA) to clean and manage the database. SPSS software (version 20.0; IBM Corp., Armonk, NY, USA) was utilized to analyze the data. Descriptive analyses were generated to describe the demographic characteristics of the study subjects. The chi-square test was used to compare risky sex behavior and healthy practices between urban and rural MSM. Multivariate logistic regression (backward logistic regression) was applied to identify the effects of residence on risky sex behavior, HIV knowledge, and healthy practices. In all models, the residence was the independent variable and risky sex behavior, HIV knowledge, and healthy practices were dependent variables, adjusted for age in years (0, ≤ 25;1, 26–40; 2, > 40), area of residence (0, Zhejiang Province; 1, Other province), education level (0, high school and below; 1, college and above), annual income (×10,000 RMB) (0, ≤ 9; 1, > 9), marital status (0, unmarried/divorced/cohabiting; 1, married/cohabiting), and sexual orientation (0, homosexual; 1, bisexual; 2, heterosexual/unsure). A *p-*value < 0.05 was considered to be statistically significant.

### Ethnics approval and consent for publication

All procedures conducted in this study were approved by the Ethics Committee of Zhejiang Provincial Center for Disease Control and Prevention (Approval Number: 2022-009-01). Electronic informed consent was obtained from all participants included in the study.

## Results

The demographic and behavior assessments included 1,993 MSM, of whom 68.6% (1,368/1,993) were aged > 25 years; 61.4% (1,223/1,993) were residents of Zhejiang Province. A total of 1,166 (58.5%) MSM had a college education and above, and 33.9% (676/1,993) reported an annual income exceeding 90,000 RMB. Among all subjects, 27.6% (551/1,993) were married or cohabiting, 60.4% (1,203/1,993) self-identified as gay, and 30.0% (597/1,993) reported being bisexual. Finally, 78.9% (1,573/1,993) of the participants lived in an urban area, and 21.1% (420/1,993) lived in a rural setting.

Compared with urban MSM, rural MSM were more likely to report Zhejiang Province as their area of residence (71.4% vs 58.7%, p < 0.001), having a high school education or below (46.2% vs. 40.2%, p = 0.028), and having an annual income ≤ 90,000 RMB (71.2% vs. 64.7%, p = 0.013). Age distribution and sexual orientation also differed significantly between urban and rural MSM according to the chi-square test results (Table 1).

**Table 1:** Social demographic characteristics of urban MSM and rural MSM (*N* = 1,993).

Variables	Total	Residence		*x* ^2^	*P*
		Urban MSM	Rural MSM		
		(N = 1,573)	(N = 420)		
		n (%)	n (%)		
Age (years)				12.484	0.002
≦ 25	625(31.4)	481(30.6)	144(34.3)		
26–40	1053(52.8)	861(54.7)	192(45.7)		
> 40	315(15.8)	231(14.7)	84(20.0)		
Permanent area of residence				22.733	<0.001
Zhejiang Province	1223(61.4)	923(58.7)	300(71.4)		
Other province	770(38.6)	650 (41.3)	120(28.6)		
Educational background				4.832	0.028
High school and below	827(41.5)	633(40.2)	194(46.2)		
College and above	1166(58.5)	940(59.8)	226(53.8)		
Annual income (×10,000 RMB)				6.197	0.013
≦ 9	1317(66.1)	1018(64.7)	299(71.2)		
> 9	676(33.9)	555(35.3)	121(28.8)		
Marital status				1.190	0.275
Unmarried/divorced/widow/widower	1442(72.4)	1147(72.9)	295(70.2)		
Married/cohabitation	551(27.6)	426(27.1)	125(29.8)		
Sexual orientation				19.312	0.000
Gay	1203(60.4)	987(62.7)	216(51.4)		
Bisexual	597(30.0)	449(28.5)	148(35.2)		
Heterosexual/unsure	193(9.7)	137(8.7)	56(13.3)		

MSM: men who have sex with men.

Of the 1,993 MSM participants, for 73.5% (1,464) the first male sexual activity was coerced, induced, or involved monetary gain. Additionally, 59.5% (1,186) and 26.9% (536) of MSM reported finding casual sex partners online and offline in the past 6 months, respectively. The proportions of MSM who ever had sex with a female had anal sex after drinking, had commercial sex, or had group sex in the past 6 months were 14.7% (293/1,993), 7.6% (151/1,993), 6.4% (128/1,993), and 4.9% (97/1,993), respectively. The proportions of MSM with inconsistent condom use with casual sex partners found online and offline in the past 6 months were 63.9% (758/1,186) and 39.9% (214/536), respectively. Stimulant use rates with casual sex partners found online and offline in the past 6 months were 40.7% (483/1,186) and 52.8% (283/536), respectively.

Of the 1,993 MSM, 80.3% (1,601) had an HIV knowledge score of 2–3. PEP and PrEP use rates were 7.1% (141) and 5.6% (111), respectively, in the past 6 months. The rate of multiple HIV tests (more than two) was 78.9% (1,573), 59.7% (1,181) of MSM reported receiving four to five kinds of HIV information, and 8.2% (163) had a high or very high risk of HIV infection.

Compared with urban MSM, a lower proportion of rural MSM found casual sex partners on the Internet (54.8% vs. 60.8%), used stimulants with online casual sex partners (33.9% vs. 42.4%) or participated in group sexual behavior (2.6% vs 5.5%). In contrast, a higher proportion of rural than urban MSM (19.8% vs. 13.4%) ever had experienced sex with a female. Compared to urban MSM, smaller proportions of rural MSM had an HIV knowledge score of 2–4 (71.4% vs. 82.7%), had undergone HIV testing more than twice (69.8% vs. 81.4%), or had used four to five types of HIV information (49.9% vs. 62.4%). Finally, a higher proportion of rural than urban MSM had high or very high awareness of HIV infection risk (14.3% vs. 6.5%). The details are listed in Table 2.

**Table 2:** Risky sexual behavior, knowledge, and healthy practices of urban and rural MSM (N = 1,993).

Variables	Total n(%)	Residence		*x* ^2^	*P*
		Urban MSM	Rural MSM		
		(N = 1,573)	(N = 420)		
		n (%)	n (%)		
**Risky sexual behavior**					
Context in which male sexual activity first occurred				10.881	0.001
Proactive and voluntarily	529(26.5)	391(24.9)	138(32.9)		
Coerced/induced/monetary gain	1464(73.5)	1182(75.1)	282(67.1)		
Finding casual sex partners online in the past 6 months	1186(59.5)	956(60.8)	230(54.8)	4.975	0.026
Usage of stimulants with online casual sex partners in the past 6 months*	483(40.7)	405(42.4)	78(33.9)	5.485	0.019
Condom use with online casual sex partners in the past 6 months* (inconsistent)	758(63.9)	608(63.6)	150(65.2)	0.211	0.646
Finding casual sex partners offline in the past 6 months	536(26.9)	412(26.2)	124(29.5)	1.872	0.171
Usage of stimulants with offline casual sex partners in the past 6 months**	283(52.8)	227(55.1)	56(45.2)	3.776	0.052
Condom use with offline casual sex partners in the past 6 months** (inconsistent)	214(39.9)	160(38.8)	54(43.5)	0.883	0.347
Sex with female ever	293(14.7)	210(13.4)	83(19.8)	10.867	0.001
Anal sex after drinking in the past 6 months	151(7.6)	124(7.9)	27(6.4)	1.001	0.317
Commercial sex in the past 6 months	128(6.4)	105(6.7)	23(5.5)	0.793	0.373
Group sex in the past 6 months	97(4.9)	86(5.5)	11(2.6)	5.808	0.016
**HIV knowledge and healthy practices**					
HIV knowledge score				26.693	0.000
0–1	392(19.7)	272(17.3)	120(28.6)		
2–3	1601(80.3)	1301(82.7)	300(71.4)		
PEP usage in the past 6 months	141(7.1)	115(7.3)	26(6.2)	0.633	0.426
PrEP usage in the past 6 months	111(5.6)	92(5.8)	19(4.5)	1.106	0.293
More than two HIV tests	1573(78.9)	1280(81.4)	293(69.8)	26.870	0.000
Number of types of HIV information obtained				21.220	0.000
0–3	796(40.3)	588(37.6)	208(50.1)		
4–5	1181(59.7)	974(62.4)	207(49.9)		
Awareness of HIV infection				26.428	0.000
No/low risk	1830(91.8)	1470(93.5)	360(85.7)		
High/very high risk	163(8.2)	360(6.5)	60(14.3)		

HIV: human immunodeficiency virus; PEP: post-exposure prophylaxis; PrEP: pre-exposure prophylaxis.

*: The denominator was MSM who finding casual sex partners online in the past 6 months;

**:The denominator was MSM who finding casual sex partners offline in the past 6 months.

Multiple logistic regression revealed relationships between residence and risky sexual behavior, HIV knowledge, and healthy practices. Compared to urban MSM, rural MSM had lower odds of finding casual sex partners on the Internet in the past 6 months (adjusted odds ratio [aOR]: 0.790, 95% confidence interval [CI]: 0.633–0.986). The odds of usage of stimulants with online casual sex partners in the past 6 months among rural MSM were lower than in urban MSM (aOR: 0.688, 95% CI: 0.506–0.936). Rural MSM had higher odds of sexual behavior with a female than urban MSM (aOR: 1.460, 95% CI: 1.036–2.058).

The odds of an HIV knowledge score of 2–3 score in rural MSM were lower than in urban MSM (aOR:0.586, 95% CI: 0.454–0.756). Compared to urban MSM, rural MSM had lower odds of having undergone more than two HIV tests (aOR: 0.583, 95% CI: 0.455–0.748) or having obtained four to five kinds of HIV information (aOR: 0.661, 95% CI: 0.529–0.826). The odds of awareness of HIV infection risk as high/very high among urban MSM were 2.312 times higher than those of rural MSM (95% CI:1.638–3.263). See Table 3 for details.

**Table 3:** Multiple logistic regression analyses of the relationships between type of residence and risky sexual behavior, knowledge, and healthy practices.

Dependent variables	aOR	95%(CI)	*P*
Risky sexual behavior			
Finding casual sex partners online in the past 6 months			
Urban MSM	Ref.		
Rural MSM	0.790	0.633–0.986	0.036
Usage of stimulants with online casual sex partners in the past 6 months			
Urban MSM	Ref.		
Rural MSM	0.688	0.506–0.936	0.017
Condom use with online casual sex partners in the past 6 months (inconsistent)			
Urban MSM	Ref.		
Rural MSM	1.118	0.820–1.523	0.481
Finding casual sexual partners offline in the past 6 months			
Urban MSM	Ref.		
Rural MSM	1.084	0.850–1.382	0.517
Usage of stimulants with casual sex partners offline in the past 6 months			
Urban MSM	Ref.		
Rural MSM	0.704	0.468–1.059	0.092
Condom use with offline casual sex partners in the past 6 months (inconsistent)			
Urban MSM	Ref.		
Rural MSM	1.305	0.850–2.003	0.224
Sex with female ever			
Urban MSM	Ref.		
Rural MSM	1.460	1.036–2.058	0.031
**HIV knowledge and healthy practices**			
HIV knowledge score of 2–3			
Urban MSM	Ref.		
Rural MSM	0.586	0.454–0.756	0.000
More than two HIV tests			
Urban MSM	Ref.		
Rural MSM	0.583	0.455–0.748	0.000
Four to five types of HIV information obtained			
Urban MSM	Ref.		
Rural MSM	0.661	0.529–0.826	0.000
Awareness of HIV infection risk as high/very high			
Urban MSM	Ref.		
Rural MSM	2.312	1.638–3.263	0.000

aOR: adjusted odds ratio; CI: confidence interval.

Adjusted for age in years (0, ≤ 25;1, 26–40; 2, > 40), area of residence (0, Zhejiang Province;1, Other province), education level (0, high school and below; 1, college and above), annual income (×10,000 RMB) (0, ≤ 9; 1, > 9), marital status (0, unmarried/divorced/widow/widower; 1, married/cohabitation), and sexual orientation (0, homosexual; 1, bisexual; 2, heterosexual/unsure).

## Discussion

Here, we used data from a large cohort study in Zhejiang Province to evaluate and compare urban and rural MSM with respect to risky behavior, HIV knowledge, and healthy practices. Our results revealed broad trends and significant differences between urban and rural MSM in Southeast China. Rural MSM has received more attention since the creation of HIV prevention programs [17]. Whether the gap between urban and rural MSM regarding HIV-related risky behavior and intervention services is getting larger or smaller has been difficult to determine.

Compared to urban MSM, rural MSM were older, native, less educated, and poorer. These differences coincided with those between rural and urban residents in the general population, as did the results regarding risky behavior and healthy practices [18]. Thus, HIV prevention among MSM is more challenging in rural areas. Studies have revealed that several demographic factors, including age, wealth index, education, and access to information have significant associations with HIV/AIDS knowledge and the availability of HIV prevention services [19, 20]. At the same time, rural MSM may face more stigma from their communities, potentially leading to anxiety, depression, and substance use/abuse [21, 22]. This is an important consideration when implementing HIV preventative services.

Regarding HIV-related risky sexual behavior, the rural MSM in this study were less likely than urban MSM to report web-based casual sexual behavior or usage of stimulants with online casual sex partners in the past 6 months. The degree of peer influence and inconvenience of offline meetings may explain the differences [23]. Compared to urban MSM, rural MSM may have access to smaller same-sex social networks [24]. Moreover, it is less convenient to meet up with casual sex partners found online in rural areas, which have fewer hotels. Rates of stimulants usage with online casual sex partners differed between the two groups; however, no differences were noted with regard to stimulants usage with casual sex partners offline. A UK study showed no difference in sexualized drug use or distance travelled for sexualized drug use between rural and urban MSM [25, 26]. This may be due to the accessibility of the drugs. In general, it is not convenient to obtain drugs related to sexual behavior in rural areas.

Furthermore, there was no significant difference between the rural and urban MSM in this study in terms of condom use with casual sex partners found online or offline. HIV behavioral skills, HIV/AIDS intervention services, and “response costs” had direct positive and negative effects on condom use [10]. Lack of access to condoms, challenges in negotiating condom use, and the preference of some for condomless sex (deemed more pleasurable) were barriers to condom use among rural MSM. This should be considered when developing HIV prevention strategies [27].

We also compared HIV knowledge and healthy practices between our two study groups. Rural MSM reported lower HIV testing rates, similar to the findings from a US study [17]. Lower HIV testing rates in rural areas may help explain this result [28]. In recent years, there has been a greater effort to improve HIV testing and accessibility for rural MSM in Zhejiang Province. Mobile testing vehicles provide weekly HIV testing to surrounding rural areas, thus partially meeting the need for testing. Rural MSM can also apply for HIV self-testing reagents online (provided by CBOs). Local hospitals provide HIV testing. However, barriers to services continue to be widespread [29]. HIV testing for MSM has always been conducted by CBOs located in urban areas. Rural MSM rarely has the choice to visit a local hospital for testing, as the doctors tend to be local residents [30]. Rural MSM reported that their communities are less tolerant toward gay and bisexual persons than urban MSM [17]. Hence, health promotion strategies and HIV-related services are needed to increase the coverage and utilization of HIV self-testing reagents, condoms, and lubricants [27]. Furthermore, a lack of HIV knowledge among rural MSM and low availability of HIV-related information highlights the need for the provision of more HIV-related information.

One interesting result of this study was that rural MSM reported higher awareness of HIV infection risk, and they exhibited less risky sexual behavior compared to urban MSM. This may be due to the lack of HIV prevention services [30]. HIV testing, as well as PEP and PrEP treatment services, tend to be located in urban areas, far from rural settings. Research has shown that long distances decrease PrEP utilization by suburban MSM [31], who tended to participate in the same risky behavior as urban MSM at parties in urban areas. The lack of key information on HIV prevention, such as PEP or PrEP treatment, as well as on the usage of stimulants, may further exacerbate the lack of awareness of risks.

In this study, we did not identify differences in PEP or PrEP use between urban and rural MSM groups; use levels (< 10%) were much lower than in other countries [32]. Overall, our findings suggest that it is crucial to improve HIV prevention services and initiate intervention programs led by CBOs in both urban and rural areas.

Our study had several important limitations. First, the convenient sampling method reduces the representativeness of the sample, especially for rural MSM. A larger sample size would have decreased potential sample bias. Second, rural residence was self-reported and may have been subject to misclassification. Moreover, groups such as suburban residents were not considered during data collection. Third, the multivariate analyses only adjusted for five variables potentially influencing the relationships between residency and the dependent variables. Future research must aim to address these weaknesses.

## Conclusion

Compared to urban MSM, the rural MSM were less likely to have engaged in web-based casual sex behavior or usage of stimulants with online casual sex partners and they were more likely to report having had sex with a female. Furthermore HIV knowledge and HIV tests number were much lower in rural MSM, who had a higher awareness of HIV infection risk. Paying more attention to HIV risky behavior and improving healthy practices in rural areas may help to prevent HIV transmission.

## Supporting information

S1 File (DOCX)
