# Supplementary material for: Urban–rural disparity in risky sexual behavior, HIV knowledge, and healthy practices among men who have sex with men: A cross-sectional study in Southeast China
Source: PLoS One. 2024 Nov 11;19(11):e0312006. doi: 10.1371/journal.pone.0312006 (PMC11554129; doi:10.1371/journal.pone.0312006)
Supplement: S1 File — (DOCX) [file pone.0312006.s001.docx]

1. Please ensure that the author list and affiliations are correct on the title page of your manuscript, and that your author contributions, competing interests, and financial disclosure are correct as listed below. All of these sections will be indexed in PubMed and published by PLOS ONE as you have written them. Please email plosone@plos.org if any changes to this content need to be made.

Wanjun Chen:

Data curation

Investigation

Methodology

Lin Chen:

Formal analysis

Writing – original draft

Writing – review & editing

Lin He:

Investigation

Chengliang Chai:

Project administration

Resources

Supervision

Please see here for the full list and definition of contributor roles: http://journals.plos.org/plosone/s/authorship#loc-author-contributions

Please ensure that the Competing Interests and Financial Disclosure statements listed below are suitable for publication. These sections will be indexed in PubMed and published by PLOS ONE as you have written them. Please email plosone@plos.org if any changes to these statements need to be made.

Competing Interests:

The authors have declared that no competing interests exist.

Financial Disclosure:

The author(s) received no specific funding for this work.

Reply: There were some revisions on author contributions

Wanjun Chen:

Data curation

Investigation

Lin Chen:

Project administration

Formal analysis

Writing – original draft

Writing – review & editing

Lin He:

Investigation

Chengliang Chai:

Resources

Supervision

2. There is a difference between the author names listed in the submission and your manuscript file.

An author is listed as Chenliang Chai in the manuscript file but as Chengliang Chai in the online submission form. If the name is incorrect in the manuscript, please correct this. If the name is incorrect in the submission form, please let us know so we can correct the author’s profile for you.

Reply: The name "Chengliang Chai" in the online submission form was correct and I have revised the name in the manuscript.

3. We note that the Competing Interests Statement given in your manuscript does not match the Competing Interests Statement given in the submission system. Competing Interests statements should not be included within the manuscript file, therefore, please remove this statement from the manuscript file and ensure that the statement is correct in the submission system.

Reply: I have removed the statement from the manuscript.

4. To prevent production delays, we recommend using the Author Formatting Checklist to confirm that your paper meets PLOS ONE's typesetting requirements for References, Tables, and Figures: http://journals.plos.org/plosone/s/file?id=c819/plos-one-author-formatting-checklist.docx.

This checklist is a reference tool for you; please do not upload the completed Author Formatting Checklist with your submission files.

Reply: I have checked all the items in the checklist.

5. To ensure your figures meet our technical requirements, please run each figure included in your submission files through the PACE tool: https://pacev2.apexcovantage.com/. PACE will assess whether your figures meet our technical requirements and will fix the figure(s) or identify any problem(s) that cannot be automatically fixed. It can also convert figures to TIFF format, resize, and rename figures to meet our naming conventions.

To use PACE, first register as a user. Follow the instructions on the site for assessing and converting your figure files. If you experience any difficulty using this tool or have questions about any of the figures and/or images in your paper, please inform the journal office in your response letter.

Reply: there is no figure.
